# Deep learning based multimodal urban air quality prediction and traffic analytics

**DOI:** 10.1038/s41598-023-49296-7

**Published:** 2023-12-13

**Authors:** Saad Hameed, Ashadul Islam, Kashif Ahmad, Samir Brahim Belhaouari, Junaid Qadir, Ala Al-Fuqaha

**Affiliations:** 1 Division of Information and Computing Technology, College of Science and Engineering, Hamad Bin Khalifa University, Doha, Qatar; 2 Department of Computer Science, Munster Technological University Cork, Cork, Ireland; 3 Department of Computer Science and Engineering, College of Engineering, Qatar University, Doha, Qatar

**Keywords:** Mathematics and computing, Computer science

## Abstract

Urban activities, particularly vehicle traffic, are contributing significantly to environmental pollution with detrimental effects on public health. The ability to anticipate air quality in advance is critical for public authorities and the general public to plan and manage these activities, which ultimately help in minimizing the adverse impact on the environment and public health effectively. Thanks to recent advancements in Artificial Intelligence and sensor technology, forecasting air quality is possible through the consideration of various environmental factors. This paper presents our novel solution for air quality prediction and its correlation with different environmental factors and urban activities, such as traffic density. To this aim, we propose a multi-modal framework by integrating real-time data from different environmental sensors and traffic density extracted from Closed Circuit Television footage. The framework effectively addresses data inconsistencies arising from sensor and camera malfunctions within a streaming dataset. The dataset exhibits real-world complexities, including abrupt camera or station activations/deactivations, noise interference, and outliers. The proposed system tackles the challenge of predicting air quality at locations having no sensors or experiencing sensor failures by training a joint model on the data obtained from nearby stations/sensors using a Particle Swarm Optimization (PSO)-based merit fusion of the sensor data. The proposed methodology is evaluated using various variants of the LSTM model including Bi-directional LSTM, CNN-LSTM, and Convolutions LSTM (ConvLSTM) obtaining an improvement of 48%, 67%, and 173% for short-term, medium-term, and long-term periods, respectively, over the ARIMA model.

## Introduction

Since the first industrial revolution in the 18th century, the planet’s environment has experienced ongoing devastation due to factory emissions and an increase in urban activities, such as vehicle traffic, mining, and farming. As a result, various pollutants are increasingly released into the environment. Air quality is one of the major concerns resulting from the deterioration of the environment and the release of pollutants. The air could be polluted by various contaminants, such as Particulate Matter (*PM*1.0), *PM*2.5, *PM*10, *CO*, $NO_2$, and $SO_2$, as determined by the US Environmental Protection Agency (EPA)^1^. Each of these pollutants is emitted into the environment due to several factors, such as traffic, industrial waste, and gaseous emissions from homes and factories. Additionally, $NO_{2}$ from agricultural waste is also a significant contributor to air pollution.

Poor air quality has a direct impact on people’s health, resulting in various illnesses, such as lung diseases, asthma, cancer, and even fatalities^2^. According to the World Health Organization (WHO)^3^, around 99% of the global population breathes air exceeding WHO guideline limits and contains high levels of pollutants where the highest exposure is observed in low- and middle-income and developing countries. According to the UN Environment program^4^, the World Health Organization’s (WHO) air quality guidelines are necessary to avoid the impacts of bad air, which causes around 7 million premature deaths per year. Policies and efforts to reduce air pollution could significantly improve air quality leading to improved climate and public health that will ultimately reduce the burden on the economy of low and middle-income countries.

Real-time monitoring and forecasting of air quality are critical to remediation by keeping the authorities informed about the air quality to take necessary precautions and make informed decisions^5^. A system able to predict air pollution in the short, medium, and long term period and learn the correlation between urban activities and air pollution could greatly help in policy-making decisions. This correlation is critical as the deployment of air pollutant sensors in all urban settlements is impractical. Moreover, data sources providing spatial and temporal correlations with air quality could create new opportunities in the domain.

Taking this into consideration, we utilized sensor data in conjunction with weather station data to develop an enhanced model capable of accurately predicting the air quality index (AQI) for short-, medium-, and long-term periods. Additionally, we leveraged the extensive coverage of CCTV cameras installed at key locations throughout the urban area to analyze the correlation between traffic volume and air pollutants. Thus, the proposed framework not only forecasts AQI but also identifies correlations between air quality and urban activities in cities and uncovers patterns.

**Figure 1:**
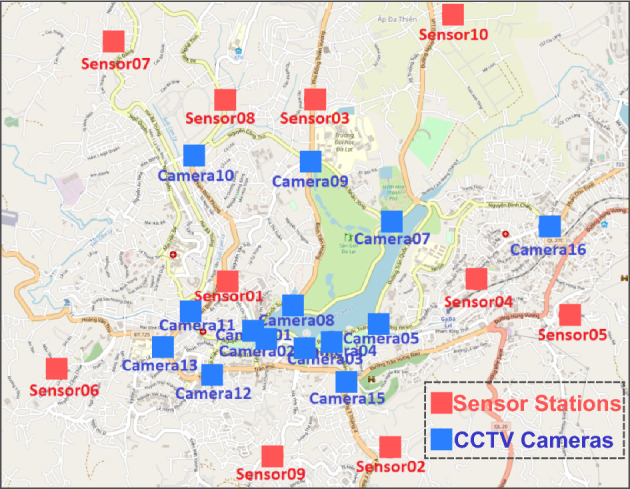
10 Air pollutants sensors and 14 CCTV cameras installed in Dalat city, Vietnam^7^.

The proposed methodology is multimodal and works with temporal and spatial data. For the temporal data, we apply Long Short Term Memory (LSTM) and its variants on real-time data on air pollutants. For the spatial data, we use the YOLO (You Only Look Once) v8^6^ object detection model to count the vehicles in the CCTV images, which is then fed into the LSTM model along with the other sensor data for predicting AQI. The models are trained on real-time air pollutant data and CCTV traffic images including continuous readings from 10 atmospheric sensors, 3 weather stations, and 16 CCTV cameras installed in various locations in Dalat City, Vietnam^7^ as shown in Fig.  1. The dataset is better suited for the application as it covers most of the real-time issues, such as sudden camera or sensor offline modes, noise from the surroundings, and outliers due to random reasons.

The AQI is predicted using both single and multiple sensors as input to the LSTM models. In the case of sensor fusion, the air quality of a certain region has been predicted using data collected from all the sensors as input to the model. This also allows forecasting the AQI for the regions where the sensors or weather station is temporarily offline. Since the sensors are placed at different locations, certain sensors will have more distance than the others from the region at which AQI is predicted. Thus, we can not treat all the sensors equally as the sensor closer to the target region may have more impact compared to the others due to similar kinds of weather and other environmental factors. Keeping this in consideration, we employed the Particle Swarm Optimization technique (PSO) to assign weights to the readings of each sensor in the average formula to make a single input window for the model.

The key contributions of the paper can be summarized as follows:

- We propose a PSO and LSTM-based multi-modal framework for accurate prediction of air quality in a region for short, medium, and long-term periods.
- A merit-based fusion of data is proposed to combine data from multiple sensors/stations, instead of relying on a single station data, to train a joint model able to accurately predict air quality at different regions. This scheme also allows the prediction of air quality in regions where the sensors are temporarily offline or do not exist.
- We also explore the correlation between air quality and urban activities that could lead to interesting applications in the future. More specifically we leverage the CCTV cameras installed at different locations in urban areas to analyze the correlation between traffic volume and air pollutants.
- We also evaluate several variants of LSTMs including Bi-directional LSTM, CNN LSTM, and ConvLSTM in the proposed framework, and compare them against two conventional time series analysis models, namely ARMA and ARIMA.

The rest of the paper is organized as follows. Section “Related Work” provides an overview of the related work. Section “Problem Statement and Motivation” specifies the problem and motivation behind pursuing this problem. Section “Methodology" describes the proposed methodology and the experimental setup. Section “Model Description” provides a comprehensive detail of the model/technique used. Section “Results” reports the conducted experiments and the experimental results. Section “Summary and Lessons Learned” summarizes the work and discusses the lessons learned during this research. Finally, Section “Conclusion and Future Work” concludes the work.

**Figure 2:**
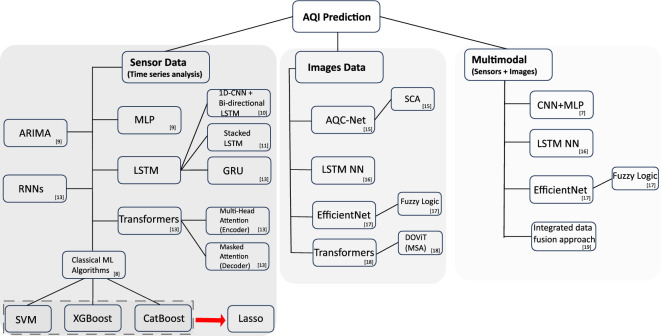
Taxonomy of different models used in AQI prediction based on sensor’s data (left), Image data (middle), and Multimodal data (right).

## Related work

The literature reports several interesting contributions toward air quality analysis. These contributions encompass a diverse array of methodologies employed for the prediction of air quality index. The framework classifying these varied approaches is visually depicted in Fig.  2 and is summarised in Table 1. The taxonomy is organized into three distinct sections, each predicated on the nature of the data employed as input for forecasting air quality. The initial section focuses on the utilization of solely sensor-derived data to predict air quality. The second section delves into the deployment of various types of imagery data for forecasting purposes. Finally, the third section pertains to the integration of both sensor-generated data and imagery to enhance the accuracy of air quality predictions.

### Sensor’s data as an input

A vast majority of the existing literature relies on sensor data for air quality prediction. For instance, Fujita et al.^8^ employed sensor data including sensors for humidity and temperature along with timestamp and location information, and public weather data for training ML models to predict air quality. They also used a stacking approach by employing three models at the first level and their outputs are then fed as inputs into other regressors at the second level. Prediciton vectors generated during training are subsequently utilized in conjunction with the test data to inform the final prediction, potentially introducing bias into the system. Liu et al.^9^ also relied on sensor data for analyzing Beijing’s AQI from 2019 to 2021. Two different models namely ARIMA and Neural Networks (NNs) are trained on the sensor data where better results are obtained with NNs compared to ARIMA. The authors recommended the suitability of sensor data for the task and concluded that the ARIMA model lags the ability to handle complex, non-linear relationships, extract relevant features, model temporal dependencies, and adapt to changing conditions making it a more unsuitable choice for forecasting AQI. Du et al.^10^ introduced the Deep Air Quality Forecasting Framework (DAQFF), which is a hybrid deep learning approach that effectively addresses the challenges of predicting urban air quality, encompassing variables like PM2.5, wind speed, and temperature. The key components of the model include one-dimensional Convolutional Neural Networks (1D-CNNs) for local trend and spatial correlation extraction and Bi-directional LSTM for learning spatial-temporal dependencies. Despite demonstrating strong predictive performance and generalization capabilities, the study’s limitations include an absence of in-depth analysis regarding computational demands and the real-time feasibility of DAQFF implementation.

In^11^, historical data on air quality and meteorological conditions are utilized for forecasting future air quality levels. The proposed approach employs a Deep spatial-temporal Ensemble (STE) model, which consists of three components. Firstly, an Ensemble learning method is employed to train different models for distinct weather patterns, as each weather pattern exhibits unique spatial-temporal characteristics in relation to air quality. Secondly, spatial correlation is investigated using the Granger Causality method^12^. Lastly, a temporal predictor is designed to capture short-term and long-term dependencies of air quality, utilizing deep LSTM (Long Short Term Memory) networks. These solutions are only based on sensor data. In the study, distinct sub-models are employed to discern spatial correlations among specific stations and areas, as opposed to our single, universal model that identifies correlations across all stations and areas where CCTV cameras have been deployed. One of the existing works^13^ evaluates four distinct deep learning approaches including RNN, LSTM, GRU, and the attention-based Transformer model, along with a baseline method on sensor data. To this aim, the Beijing Air Quality Dataset is employed for experimentation, comprising multivariate hourly time series data spanning five years. The findings in this research demonstrate the superior predictive capabilities of the Transformer model when forecasting up to 4 hours ahead. Subsequently, from the 4th to the 16th hour, the performance of LSTM aligns closely with that of the Transformer model. This trend underscores the Transformer’s proficiency in predicting early hours, particularly within the 4 to 5-hour range. While Transformers have exhibited promise in numerous Natural Language Processing (NLP) applications, they encounter substantial difficulties when applied to long sequence time-series forecasting^14^. These challenges arise from their quadratic computational complexity in self-attention, memory limitations when stacking layers for extended inputs, and a decrease in inference speed when generating lengthy outputs, collectively impeding their efficiency in handling prolonged sequences. Notably, our study extends beyond this time frame, encompassing predictions for more distant futures such as 5 days and 10 days ahead. Zhang et al.^15^, present a lightweight approach for PM2.5 prediction utilizing a novel model called Sparse Attention-based Transformer Networks (STN) for both single-step and multi-step forecasting scenarios. The study emphasizes the necessity of addressing sudden fluctuations in air pollution data to enhance forecasting accuracy, crucial for environmental protection and public health. In the study, the future forecasting spans 48 hours, whereas our study extends predictions to 192 hours ahead. Although the results from STN model align with our outcomes, it’s essential to mention that the STN model exhibits higher complexity compared to our simpler LSTM variants, emphasizing the simplicity and ease of implementation in our proposed approach.

**Table 1:** Short description and limitations of the research work done in the field of Urban Air Quality prediction and correlation with Urban nature activities.

Input data type	Reference	Year	Short description	Limitations
Sensor’s data	^8^	2021	Stacking approach of different ML models has been used for predicting air quality	Prediction vectors generated during training are used with test data for final prediction thereby, introducing bias into the system
	^9^	2022	ARIMA and Neural networks are trained and results are compared	Models applied lags the ability to handle complex, non-linear relationships and adapt to changing conditions
	^10^	2019	Hybrid DL approach used to predict Urban air quality using 1D-CNNs and Bi-directional LSTM	Absence of in-depth analysis regarding computational demands and the real-time feasibility of the model
	^11^	2018	Deep spatial-temporal Ensemble (STE) model used for extracting distinct weather patterns, spatial correlation and short-term and long-term dependencies of air quality	Distinct models used for extracting spatial and temporal dependencies for different stations compared to our single model across all the stations
	^13^	2022	Four distinct DL approaches i.e. RNN, LSTM, GRU, and Transformer model used for forecasting air quality. Every 4-hour window is being forecasted for the next 48 hours	Results showed that from the 4th to 16th hour of forecasting, the performance of LSTM aligns with the Transformer model. Our work also shows results for the next 5 days and 10 days forecasting
	^15^	2023	The study stresses the importance of addressing air pollution fluctuations for accurate PM2.5 forecasts using a lightweight method, STN, in single-step and multi-step scenarios	The range of future prediction is 48-hours as compared to ours 192-hours. Also STN model is more complex than our simpler LSTM variants, underlining the ease of implementation in our approach
Images Data	^16^	2020	A self-supervision module called Spatial and Context Attention (SCA) block feature representation between channel maps.	The dataset used in this paper is limited to only day time focusing more on sky-dominated scenes
	^17^	2021	Set of patterns using transfer learning, fuzzy negation, and periodic frequent pattern mining establishing a correlation between urban nature, traffic, and air pollution	The data acquired in this paper represents collective average values encompassing all regions of the city as compared to our weighted averaging approach for data of different regions
	^18^	2022	Double Output Vision Transformer (DOViT) structure is used for automatic feature extraction from the images for prediction of air quality	Data mostly comprises of sky images, buildings and scenic images as compared to our CCTV traffic images captured in real-time
Multimodal Data	^19^	2021	Mobile images used with certain hypothesis regarding correlation of air quality with urban nature. LSTM-NN model has been used for prediction	The image dataset used does not consider traffic impact on air quality, instead, it is based on the general environment i.e. scenic images to predict
	^17^	2021	Semantic Segmentation is used to segment urban nature segments from images like buildings, sidewalks, and minibikes. The ratio of each category is used along with the air quality levels to predict	Mobile images are used instead of CCTV images provided by the authorities. Only 100 images are used in one iteration to calculate precision. Smaller data does not cover all the scenarios of the urban nature. Moreover, the accuracy is lower as compared to our achieved accuracy
	^20^	2019	Numerical data plus visual data (Environmental pictures) are fed into a system of CNNs and MLP to predict air quality index	Accuracy was found to be low. Also, forecasting of air pollutant values are not done instead, the system is trained to predict AQI level directly which means the previous calculation towards the AQI levels has been done manually
	^21^	2022	Satellite images along with the sensors data are used to enhance the accuracy for assessing air quality	This study is limited only to satellite images
	^22^	2023	A deep learning model utilizing an encoder-decoder architecture and real-time monitoring data. This model predicts PM2.5 concentration and evaluates the impact of urban traffic on air quality	Their prediction timeframe was limited to 48 hours, which is significantly shorter than our extended prediction range of 192 hours.

### Images as an input

The literature also reports the use of image data for air quality prediction. For instance^16^, introduces AQC-Net, a deep learning-based model for air quality estimation from scene images. A self-supervision module called Spatial and Context Attention (SCA) block enhances feature representation by capturing interdependence between channel maps. Experimental results on the NWNU-AQI (a high-quality multi-scenario air quality) dataset^16^ demonstrate superior air quality classification accuracy compared to SVM and ResNet methods, addressing the critical need for effective air quality monitoring. In this work, the utilization of exclusively daytime images, predominantly comprising sky-dominated scenes, imposes certain limitations on the applicability of this research. Furthermore, it is important to note that this work falls short of achieving the same level of monitoring accuracy as traditional air quality monitoring stations. In^17^, PM2.5 is predicted for a short and medium-term period through images captured by smartphones and cameras. A set of patterns is established by leveraging the correlation between urban nature, traffic, and air pollution using data collected from two different countries through transfer learning, fuzzy negation, and periodic frequent pattern mining. The authors assert that their proposed methodology has the potential to facilitate the development of sustainable smart cities that prioritize the demands of urban traffic, urban management, and citizen health. The data collection process in this research involved affixing personal devices onto motorcycles and operating them along predetermined routes for extended periods. As a result, the data acquired represents collective average values encompassing all regions of the city in contrast to our study, which employs a weighted averaging approach for the data. The work also exhibits the limitation of not addressing area-specific predictions within the city. Wang et al.^18^ also utilized mobile devices to capture images and employed a Double Output Vision Transformer (DOViT) structure with a multi-head self-attention (MSA) mechanism for automatic feature extraction from the images for the task. In contrast to the existing methods, in this work, we utilize the existing CCTV traffic images captured in real-time for determining the traffic intensity by detecting vehicles and extracting the vehicle count from the images. The vehicle count feature is then provided to the proposed model for forecasting the AQI for short-term, medium-term, and long-term periods.

### Multimodal input

The literature also reports several works utilizing both sensor data and images for air quality prediction. For instance^19^, provided both sensor data and images as input to an LSTM-based model for predicting air quality. The authors reported better results for the fusion of images and real-time open PM2.5 measurements. The research work in^17^ also utilized images and sensor data for exploring the correlated features of urban nature, transportation, and environment. The study in^20^ generated AQI rankings/categories using numerical data collected from different sensors and visual data (Environmental pictures). Subsequently, the same AQI values and visual features extracted through Convolutional Neural Networks (CNNs) were fed into a multi-level perception (MLP) model to predict AQI levels in a region. The experiments were conducted on data logs obtained from five distinct routes in Tokyo, Japan. However, the accuracy of the results was found to be low, and no forecasting of air pollutant values was performed. Li et al.^21^, on the other hand, integrated three different types of information for the generation of full coverage Aerosol Optical Depth (AOD) maps to enhance the overall data accuracy for assessing air quality and pollutant concentrations in China. The data sources include satellite AOD data, daytime AOD snapshots, and other sensor data. The method used here is an “integrated data fusion approach”. Instead of using observational data solely as learning targets, the proposed method fuses multimodal AODs derived from multiple data sources. The study is limited to satellite imagery. Dao et al.^19^, employed open datasets containing multimodal data including air pollution, weather, and image data, which are used for training ML models for air quality prediction. The authors proposed a method for predicting AQI at a local and individual scale using a few images captured from smartphones and open AQI and weather datasets, leveraging lifelog data and urban nature similarity. Moreover, a Conditional Long Short-Term Memory Neural Network (LSTM-NN) model has been used for the prediction. The experimental results revealed that using only PM2.5 values as an input to the model for prediction yielded poor results while using solely images to forecast PM2.5 levels led to satisfactory results. Historical images captured within the same geographical context, such as a specific route or location, along with the extraction of high-semantic features from these images, have the capability to serve as predictive indicators for PM2.5 concentrations.

The literature shows several emerging challenges in understanding the mutual impact of air pollution and urban life through monitoring systems consisting of sensor data and CCTV images. The availability of data for research is highlighted as a significant barrier for researchers^7^. Existing datasets used by researchers are often limited in size and spatiotemporal dimensions. They may also lack realistic conditions, such as missing, noisy, or outlier data. This work is based on real-time data, provided in a benchmark competition^7^, reflecting real-world conditions and issues, such as sudden camera or station malfunctions, noise in the data, and outliers. The model trained on data collected from multiple stations in an optimized way not only improves the performance of the framework but also allows the prediction of air quality in regions where the sensors are temporarily offline or do not exist. Moreover, the vehicle count feature extracted from the provided CCTV imagery further enhances the predictive efficacy of our model. In the cited paper^22^, researchers proposed a deep learning model named iDeepAir employing an encoder-decoder architecture and real-time monitoring data to predict PM2.5 concentration and assess the influence of urban traffic on air quality. However, their prediction timeframe was limited to 48 hours, significantly shorter than our extended prediction range.

## Problem statement and motivation

This work aims to address some of the key limitations of the existing works, especially the data used for the prediction. The prior work mostly relied on datasets either collected from offline sources or generated within laboratory settings, which do not fully capture the complexities of real-world scenarios. To overcome these drawbacks, we leverage real-time data that is continually obtained from sensors installed in different regions of the city. Typically, weather stations and air pollutant sensors are strategically positioned within urban areas to monitor pollution levels. These sensors allow us to forecast air quality for different regions in the cities. However, in such infrastructure, sensor failures may occur, resulting in the inability to calculate air quality for a particular region. To this aim, we train a single model on data obtained from the nearby stations/sensors to further enhance the capabilities of the proposed solution. This approach allows the prediction of air quality in areas where sensors are either absent or malfunctioning during a specific time interval. Moreover, keeping in view the impact of urban activities (e.g., vehicle traffic) on the environment, we also explore the potential correlation between urban activities and air quality by incorporating the CCTV images into the model.

Notably, our study explores two key aspects. Firstly, we incorporated vehicle count as a feature extracted from CCTV images, thereby enhancing the predictive capabilities of our model and exploring the correlation between urban activities and air quality. Secondly, we implemented a merit-based sensor fusion scheme, enabling simultaneous training of the model using both vehicle count from CCTV images and air pollutant data from multiple sensors installed at different locations.

**Figure 3:**
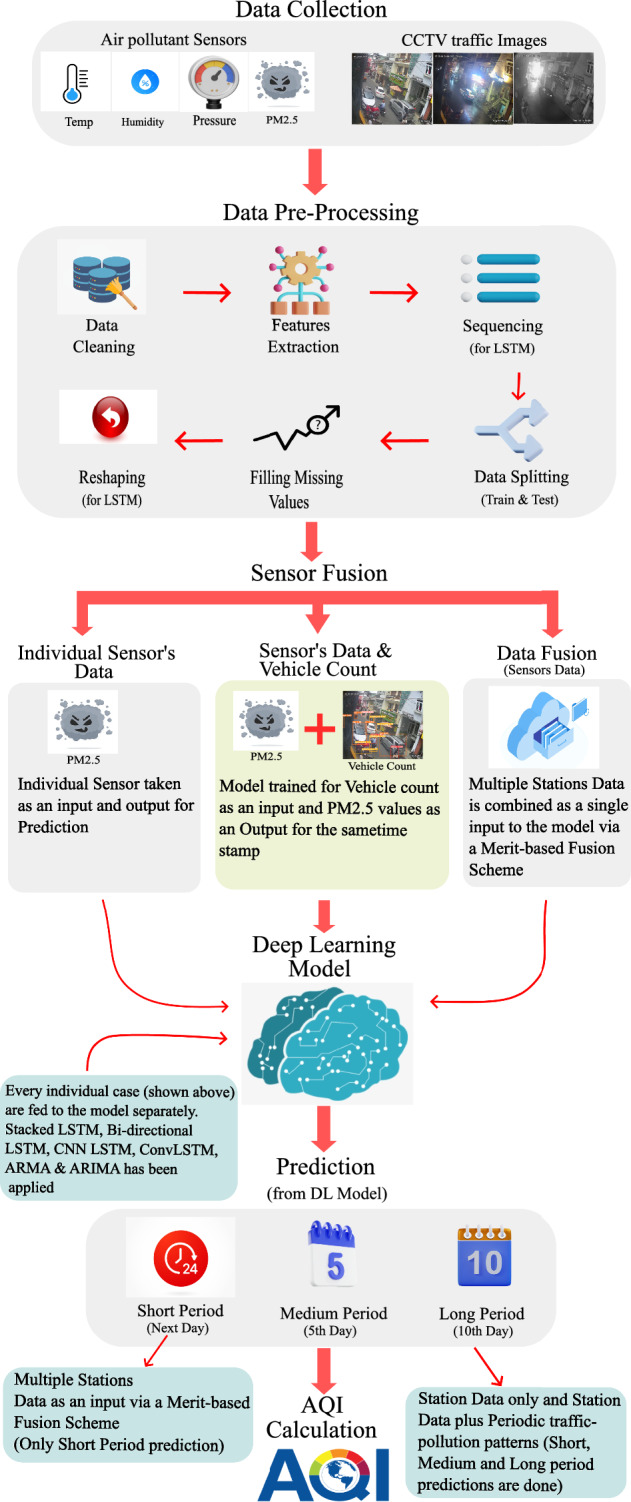
Flow Chart depicting all the major blocks of our experimental setup. Data Collection: from sensors to CCTV images^7^, Data Pre-Processing and cleaning, Data Fusion (exclusively for sensor’s data), Our Proposed Deep Learning Model, Prediction Results for Short, Medium, and Long-term Periods, and AQI Calculation.

## Methodology

An overview of the proposed methodology is provided by Fig.  3, which can be roughly divided into six phases comprising Data Collection (Phase 1); Data Pre-Processing (Phase 2); Sensor Fusion (Phase 3); training the Deep Learning Model (Phase 4); Prediction (Phase 5); and AQI calculation (Phase 6).

- In the *first phase*, multi-modal real-time data (including CCTV camera imagery and air pollution data) from the different regions of Dalat City, Vietnam is collected. The data sources include 10 sensors for air/atmospheric pollutants (*PM*1.0, *PM*2.5, *PM*10, $SO_2$, $NO_2$, *CO*, $O_3$)^7^; 3 meteorological stations providing weather data (*Temperature*, *Humidity*, *Rainfall*, *UV*)^7^ both having a refresh rate of 5 minutes which caters for any change in the wind speed, pressure and dew point; and 16 CCTV cameras for traffic images^7^ which records one frame every 5 seconds. There are two key challenges associated with this data collection process: (1) the data’s inconsistency resulting from occasional sensor or camera failures in real-time, with data collection resuming after maintenance by the authorities, and (2) the limited nature of the data, as our analysis relies on just 5 months’ worth of data with some missing values.
- In the *second phase*, we employed some pre-processing techniques to clean the data, extract features from the data, prepare the data to feed it into the model and deal with the missing values.
- In the *third phase*, we arrange three types of input data based on our objectives. Firstly, we arrange a single sensor’s data for training the model to build our base model. Secondly, we do sensor fusion and combine the input data for all the sensors and feed it into the model for training. In this case, we want the model to predict any specified region based on the combined input of all the sensors. The third scenario deals with the model being trained on both the sensor’s data and the vehicle count obtained from the CCTV camera images. Here our model takes the CCTV images as input and predicts the PM2.5 levels. In this case, we make some interesting insights from the correlation between urban traffic and urban air quality.
- In the *fourth phase*, the input data, based on the aforementioned three cases, is fed into the Deep Learning Model for training purposes. In our study, we used LSTM and its variants as our models for forecasting air quality. LSTM is chosen based on its proven performance in similar applications^23,24^.
- In the *fifth phase*, the model predicts short-term, i.e. the next day in the future, medium-term, i.e. the fifth day in the future, and long-term periods, i.e. the 10th day in the future.
- In the *sixth phase*, AQI is computed based on the model’s predictions of PM2.5 levels according to the US EPA^1^ standards.

In this work, our objectives are twofold: AQI Prediction and Pollution-Traffic Correlation Discovery. An overview of these objectives is provided below.

- *Objective 1* (AQI Prediction): Firstly, we aim to predict future values of pollutants and forecast AQI for a given area based on the data collected from the sensor/sensors installed in that area. Let’s suppose *D* is the current day. So, we predict the values of air pollutants and forecast AQI for *D*+1st day, i.e., the next day (short-term period). Similarly, the same process is repeated for the *D*+5th day (medium-term period) and *D*+10th day (long-term period). We also make use of data fusion at the input taken from multiple sensors installed at different stations to predict air quality for a target station, for the next day, i.e., for *D*+1st day.
- *Objective 2* (Pollution-Traffic Correlation Discovery): Secondly, we intend to develop a model that incorporates both the sensors’ data and CCTV images to establish a correlation between air pollution and traffic in the city. This will enable the model to predict pollutant values when given the vehicle count obtained from the CCTV images of the traffic as an input. In this task, we will predict the air quality for the same *D*+1st, *D*+5th and *D*+10th day.

All these objectives bring some variations in the pre-processing, data preparation, and fusion processes. Thus to make it more readable, this section is divided into two subsections each describing the methodology/processes for each experiment conducted in this work.

### Objective 1 (AQI prediction)

In this section, we will address two distinct scenarios. In the first case, we aim to forecast the AQI for a particular region by relying solely on the sensor deployed within that specific region. In the second case, we delve into the application of fusion techniques, synthesizing a unified input derived from all the sensors distributed across the city. With this consolidated data, we proceed to predict the AQI for any specific area, even in cases where a dedicated sensor may not be present. We provide a comprehensive and separate explanation of each scenario below.

#### AQI Prediction using the Station Data only

In this experiment/task, we want to predict air quality using station data (i.e., the sensors installed in the same area) only. To complete the task, we outline the operational procedures for one of the stations, which can be replicated for the other stations. To this aim, we have chosen station/sensor 3 due to two factors. Firstly, it is one of the sensors connected to the weather stations. Secondly, it contains more recorded values compared to the other sensors and therefore has fewer instances of missing data. For AQI calculation, each pollutant value is used separately and the one which gives the largest value of AQI is considered the one responsible for higher AQI^1^. Here, we will consider PM2.5 values and the same can be extended to other air pollutants. One justification for selecting PM2.5 air pollutants pertains to the air quality categorization and public notifications based on both AQI and PM2.5 concentration levels.

We collected data for sensor 3 which is connected to one of the weather stations for a period of 5 months, from June 15, 2022, to November 14, 2022, resulting in a total of 29,047 samples. To use this data as input for our LSTM model, we performed some pre-processing operations on the PM2.5 samples. These operations can be extended to other pollutant samples to determine the contribution of each pollutant to the AQI. The initial input matrix size is 29047x1. However, after pre-processing, which involves averaging every 5-minute sample over an hour to obtain an hourly sample, the size of the input matrix changes to 153x24. In this new matrix, each row represents the number of days while each column represents every hour of the day.

The data is arranged in a sequence based on the key variables’ *hour count*, *day count*, and *lookahead*, which will be used as input to our LSTM model. For instance, we took *hour count* = 3, *day count* = 4, and *lookahead* = 1, the model will predict the PM2.5 values for the next day at the same hours as the input hours, where the input hours will be from the previous 4 consecutive days. It is then transformed (reshaped) into a 3-dimensional matrix for the input of the LSTM model. The data is then split into training data (19 weeks) and test data (3 weeks). Due to some errors/faults in the sensor data, there are missing values, which are replaced by the mean data of the corresponding column (corresponding hour) of the training data. Both the training and test data are normalized using Eq. (1) to make it in the range of [0, 1], before training the model because the PM2.5 value range for different sensors are different (minimum recorded value for PM2.5 is 0 and maximum recorded value is 1688). Data normalization ensures equitable treatment of all features, thereby enhancing the efficiency, stability, and effectiveness of the training process. Consequently, this practice contributes to the improvement of model performance and its ability to generalize effectively to unseen data.

1 $$\begin{aligned} Xs = (X - X_{min})/(X_{max} - X_{min}) \end{aligned}$$

#### AQI Prediction using Multiple Stations Data via a Merit-based Fusion Scheme

In this task, we aim to utilize multiple stations’ data for air quality prediction in a particular region by combining the sensor data in a merit-based fusion. The main motivation for the task comes from the fact that air pollutant sensors can not be installed everywhere in the city. Also, the sensors can get faulty due to random reasons. Our objective is to find the air quality of those regions using the sensors installed and working in the nearby regions. To this aim, we performed sensor fusion by taking six sensors’ weighted averages as an input for our model and obtained results for all the individual sensors taken as an output. Using Haversine Formula 2, we discarded the data from sensor 2, sensor 4, sensor 5, and sensor 8 as the distances calculated for these sensors gave erroneous and illogical values. In the equation, $\varphi$ is latitude, $\lambda$ is longitude, *R* is earth’s radius (mean radius = 6371 km) and the angle must be in radians to use it in the trigonometric function.

2 $$\begin{aligned} \begin{aligned} a&= \sin ^{2}\left( \frac{\Delta \varphi }{2}\right) + \cos \varphi _{1} \cdot \cos \varphi _{2} \cdot \sin ^{2}\left( \frac{\Delta \lambda }{2}\right) \\ c&= 2 \cdot \arctan 2\left( \sqrt{a}, \sqrt{(1-a)}\right) \\ d&= R \cdot c \end{aligned} \end{aligned}$$

We used sensor 1, sensor 3, sensor 6, sensor 7, sensor 9, and sensor 10 in the fusion and took the same sensors as an output one by one to predict the air quality in the regions where these sensors are installed.

For this task, we used the same input window as we used in the case of AQI prediction for station data only. The only difference is the weighted average values in the input window for the fusion. First, we arranged all the data of all six sensors for the same dates and time in order to get their average values. We can not directly average all the values because each sensor may contribute differently and we need to know which of the sensors is contributing more to the combined sensor data. For this purpose, we initially calculated the distances of all the input sensors/stations from the output sensor/station and took the ratio of these distances as the weights for the weighted average. However, this is not an optimal solution, thus, to obtain near-optimal values/weights, we employed an optimization method namely Particle Swarm Optimization (PSO) for the merit-based weights to be used in the Weighted Average Formula 3. These weights result in the minimum error between the average value obtained and the real value of the output sensor.

3 $$\begin{aligned} \begin{aligned} \overline{W} = \omega _{3}.S_{3}+\omega _{7}.S_{7}+\omega _{10}.S_{10}\\ +\omega _{1}.S_{1}+\omega _{6}.S_{6}+\omega _{9}.S_{9} \end{aligned} \end{aligned}$$

In the above equation, $\overline{W}$ shows the weighted average after fusion of the sensors, $\omega _{i}$, represents the weights updated by PSO, and $S_{i}$, represents the respective sensors for which the weights are being updated, where, $i = {3,7,10,6,1,9}$. Once the input matrix of weighted average values is obtained, the same process used in the case of AQI prediction for station data only is repeated to predict the PM2.5 values for the next (*D*+1st) day (short period).

### Particle swarm optimization

Particle Swarm Optimization (PSO) is a powerful optimization technique inspired by the collective behavior of bird flocking or fish schooling and has been already proven very effective in fusion^25^. In PSO, a group of particles (representing potential solutions) traverse the search space, iteratively adjusting their positions based on their own experience and the experience of their neighbors. Each particle is influenced by its personal best solution found and the best solution discovered by its neighbors. This cooperative behavior enables PSO to efficiently explore the solution space and converge towards promising optima.

In our case, the number of particles is 6, and the upper bound is set to 1 and a lower value is approximately equal to zero, ensuring the weights in the range of 0 to 1. Our goal is to tune/optimize the weights for the Eq. (3) with the aim of aligning the resulting data matrix with the data matrix of the sensor, which is considered the output to be predicted. For instance, if we want to predict the air quality at station 3 (i.e., the region where sensor 3 is installed) while having a combined input of all the sensors then our target value in the objective function of the PSO algorithm will be based on the sensor 3 data matrix.

**Figure 4:**
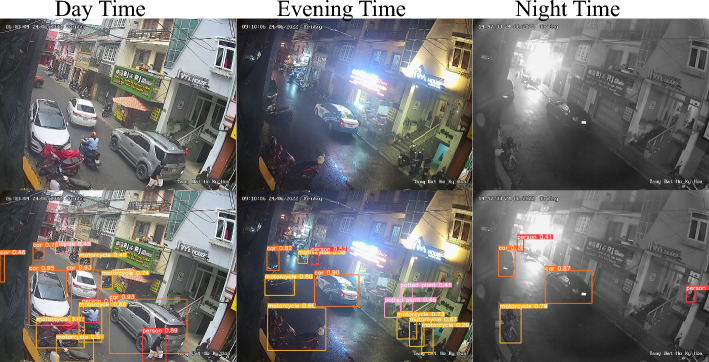
YOLOv8x weights used with the COCO dataset^26^ to detect cars, motorbikes, and buses (labels used from the COCO dataset) in the CCTV images^7^. Vehicle count from this detection is used for the discovery of Periodic traffic pollution patterns.

### Objective 2 (periodic traffic-pollution patterns discovery)

In this objective, our aim is to investigate the correlation between human activities and AQI. Additionally, we seek to explore the potential of utilizing CCTV cameras as an alternative approach to forecast AQI. We intend to establish a relationship between CCTV camera images and sensor data by incorporating CCTV traffic images as inputs to our model for AQI prediction. In this specific case, we used sensor 3 as the test case and camera 9, as camera 9 is the CCTV traffic camera closest to sensor 3, as shown in Fig. 1. Figure 1 shows the location of 10 air pollutant sensors and 14 cameras installed in the region.

In this task, our method also involves counting/identifying the number of vehicles in the CCTV images and using it as one of the features to find the correlation between traffic density and air quality. Camera 9, which is the closest to sensor 3, is selected to obtain CCTV traffic images during the same period as sensor 3 data (June 15, 2022 - November 14, 2022). However, we observed that some images were missing in July and in other months, resulting in a total of 22641 images. The images are available in two formats including *colored images* and *grayscale images*. Colored images cover daytime and evening time while grayscale images cover nighttime. To process the images, we needed a model that could tackle both types of images without compromising the results of the object detection algorithm. It is worth mentioning here that the images collected from the CCTV cameras record frames every 5 seconds and range from June to November. This dataset caters to all kinds of weather conditions i.e. Rainy and sunny weather conditions. We trained our model on this dataset having different types of weather conditions which makes our system more robust.

Initially, we employed YOLOv5^27^, which failed to produce accurate results in terms of vehicle detection in both types of images. We then used the latest YOLOv8 model, which is a larger model with a higher number of parameters. The model is available in different configurations. We tried different versions of the model including the YOLOv8n, which is the simplest among the version 8 models having 3.2 million parameters and 8.7 billion FLOPs. However, the results were not very accurate. Finally, we used YOLOv8x weights with the COCO dataset^26^ to detect objects, such as cars, motorbikes, and buses in the images, the details are provided in Table 2. YOLOv8x has 68.2 million parameters and 257.8 billion FLOPs. The model produced much better results.

**Table 2:** Total vehicle count extracted from CCTV traffic images (June 15 - Nov 14, 2022).

Total vehicle count for camera 9	
Cars	24232
Motorbikes	107153
Buses	1648
Total	133033

**Figure 5:**
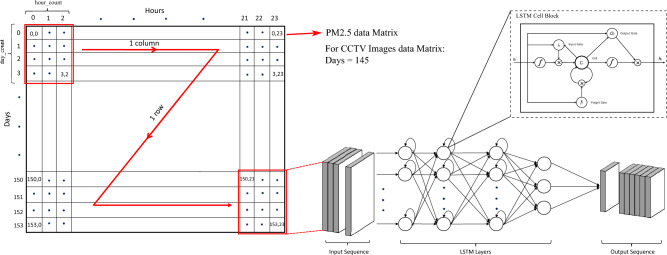
General Description of a Data Matrix sequencing. Frames are generated in sequential order for Input to our LSTM model through a windowing process shown by the red arrow line. Variable hour count covers 24 hours, day count can be any no. of days as input, and Lookahead can be any day in the future. Output sequence is generated by the LSTM layers for the desired future period.

Figure 4 shows some sample CCTV images and the corresponding output images with vehicles being detected by the model. The figure includes the images captured in the daytime, evening time, and nighttime (in the top row). The bottom row shows the output after the model detects the required objects in these images. In Table 2 it can be observed that more than 80 percent of the vehicles are MotorBikes.

In our first objective (i.e., AQI prediction), the input matrix for the model has one feature (i.e., air pollutant values) while in this case the model is trained on two types of features including the air pollutant values and the number of vehicles in the images. The same pre-processing steps used in the tasks of AQI prediction are also applied here after getting the total count of the vehicles from all 22641 images. The input matrix size changed from 22641x1 to 145x24 where 145 shows the number of days and 24 shows the number of hours. Here the total count of vehicles for every hour of every day has been calculated. All the image data is arranged according to the same sequence as the air pollutants data using the timestamp. The model is trained on both the air pollutants data and the vehicle count obtained from the CCTV images data where the latter data is used as an input to the model while the former data is taken as an output using the same key variables (*hour count*, *day count* and *lookahead*) of the generic model used in the tasks of AQI prediction.

### Model description

Figure 5 provides an overview of the proposed model. Three main variables are defined in the model including *hour count*, *day count*, and *lookahead*. The *hour count* variable represents the number of hours as an input, which ranges from 1 to 24. The *day count* variable determines the number of days in the input while *lookahead* variable allows the model to predict the desired day in the future. Each window in the model is determined by the values assigned to variables *hour count* and *day count*. Figure 5 illustrates the movement of the window across the entire data matrix, serving as an input to the LSTM model.

The general form of an element in the input window is $h^d_{ij}$, where *h* is the hourly value of the air pollutant (in this case PM2.5) determined by *i*, and *j*, *d* is the day number and *i* & *j* represents the starting time and end time of the hour, respectively. The input window for the case of *hour count* = 3, *day count* = 4 and *lookahead* = 1, will be:

$$\begin{aligned} \begin{bmatrix} h^0_{01}&{}h^0_{12}&{}h^0_{23}\\ h^1_{01}&{}h^1_{12}&{}h^1_{23}\\ h^2_{01}&{}h^2_{12}&{}h^2_{23}\\ h^3_{01}&{}h^3_{12}&{}h^3_{23}\\ \end{bmatrix} \end{aligned}$$

Our objective is to forecast the air pollutant values for the next day which is represented as *D*+1st day, where *D* is the current day, *D*+5 is the 5th day and *D*+10 is the 10th day. The output window sequence for *D+1*st day is:

$$\begin{aligned} \begin{bmatrix} h^4_{01}&{}h^4_{12}&{}h^4_{23}\\ \end{bmatrix} \end{aligned}$$

for *D+5*th day is:

$$\begin{aligned} \begin{bmatrix} h^8_{01}&{}h^8_{12}&{}h^8_{23}\\ \end{bmatrix} \end{aligned}$$

and for *D+10*th day is:

$$\begin{aligned} \begin{bmatrix} h^{13}_{01}&{}h^{13}_{12}&{}h^{13}_{23}\\ \end{bmatrix} \end{aligned}$$

In the next iteration, the input window will add the next column of the hours according to the same *hour count* for the same days and the first column of the initial input window will be removed from the input window as shown below:

$$\begin{aligned} \begin{bmatrix} h^0_{12}&{}h^0_{23}&{}h^0_{34}\\ h^1_{12}&{}h^1_{23}&{}h^1_{34}\\ h^2_{12}&{}h^2_{23}&{}h^2_{34}\\ h^3_{12}&{}h^3_{23}&{}h^3_{34}\\ \end{bmatrix} \end{aligned}$$

and the output window will also take the form for *D+1*st day:

$$\begin{aligned} \begin{bmatrix} h^4_{12}&{}h^4_{23}&{}h^4_{34}\\ \end{bmatrix} \end{aligned}$$

for *D+5*th day:

$$\begin{aligned} \begin{bmatrix} h^8_{12}&{}h^8_{23}&{}h^4_{34}\\ \end{bmatrix} \end{aligned}$$

and for *D+10*th day:

$$\begin{aligned} \begin{bmatrix} h^{13}_{12}&{}h^{13}_{23}&{}h^4_{34}\\ \end{bmatrix} \end{aligned}$$

Once all the hourly values for the same days are traversed in the input sequences the window will shift to the next row and the first row will be removed from the input window. The input window after 22 iterations for predicting *D+1*st day will be:

$$\begin{aligned} \begin{bmatrix} h^1_{01}&{}h^1_{12}&{}h^1_{23}\\ h^2_{01}&{}h^2_{12}&{}h^2_{23}\\ h^3_{01}&{}h^3_{12}&{}h^3_{23}\\ h^4_{01}&{}h^4_{12}&{}h^4_{23}\\ \end{bmatrix} \end{aligned}$$

and the output sequence for *D+1*st day will be:

$$\begin{aligned} \begin{bmatrix} h^5_{01}&{}h^5_{12}&{}h^5_{23}\\ \end{bmatrix} \end{aligned}$$

The same type of sequences can be made for the *D+5*th day and *D+10*th day.

These input sequences are fed into the proposed model and the required output is recorded. The model consists of three connected layers of LSTM blocks (known as stacked LSTM) with **’relu’** as the activation function (it helps address the vanishing gradient problem and can accelerate convergence during training). The first layer consists of 100 LSTM blocks while the second and the 3$^{rd}$ layers consist of 64 and 50 LSTM blocks, respectively. The LSTM block comprises four essential elements: an input gate, a forget gate, an output gate, and a cell state.

4 $$\begin{aligned} i_t = \sigma (W_i.[h_{t-1}, x_t] + b_i) \end{aligned}$$

The input gate determines the information to be incorporated into the cell state as per Eq. (4), while the forget gate determines the amount of information to be discarded according to the Eq. 5.

5 $$\begin{aligned} f_t= & {} \sigma (W_f.[h_{t-1}, x_t] + b_f) \end{aligned}$$

6 $$\begin{aligned} o_t= & {} \sigma (W_o.[h_{t-1}, x_t] + b_o) \end{aligned}$$

The output gate regulates the transfer of information to subsequent time steps or the output and is calculated as Eq. 6. The cell state is the result of combining the previous internal memory state and the forget gate, as well as the element-wise multiplication of the self-recurrent state and the input gate.

7 $$\begin{aligned} c_t= & {} f_t \odot c_{t-1} + i_t \odot {\sim }{c}_t \end{aligned}$$

8 $$\begin{aligned} {\sim }{c}_t= & {} ReLU(W_c.[h_{t-1}, x_t] + b_c) \end{aligned}$$

9 $$\begin{aligned} h_t= & {} o_t \odot ReLU(c_t) \end{aligned}$$

where $h_t$ is the hidden state of the LSTM block. $W_i$, $W_f$, and $W_o$ are weight matrices associated with the input gate, forget gate, and output gate respectively. ReLU is the function when it receives any negative input, it transforms that input to zero “0”, while it keeps the positive value *x* as it is. This function ranges from 0 to infinity:

10 $$\begin{aligned} f(x) = max(0, x) \end{aligned}$$

The input window sequence and the output window sequence for Objective 2 during the training and testing process have taken the following form:

Let $vc^d_{ij}$ represent the vehicle count (where *v* shows the vehicle count value for a certain hour determined by *i* and *j*) and let $h^d_{ij}$ represent the hourly value of the air pollutant (PM2.5), for a specific hour (determined by the value *i* and *j* ) and for a certain day *d*. The input window for the case of *hour count* = 3, *day count* = 4 and *lookahead* = 1, will be:

$$\begin{aligned} \begin{bmatrix} vc^0_{01}&{}vc^0_{12}&{}vc^0_{23}\\ vc^1_{01}&{}vc^1_{12}&{}vc^1_{23}\\ vc^2_{01}&{}vc^2_{12}&{}vc^2_{23}\\ vc^3_{01}&{}vc^3_{12}&{}vc^3_{23}\\ \end{bmatrix} \end{aligned}$$

Our objective is to forecast the air pollutant values for the short period which is represented as *D*+1st day, where *D* is the current day, the medium period represented as *D*+5th day and long period represented as *D*+10th day. So, the model is trained for the output window sequence as follows:

for *D+1*st day:

$$\begin{aligned} \begin{bmatrix} h^4_{01}&{}h^4_{12}&{}h^4_{23}\\ \end{bmatrix} \end{aligned}$$

for *D+5*th day:

$$\begin{aligned} \begin{bmatrix} h^8_{01}&{}h^8_{12}&{}h^8_{23}\\ \end{bmatrix} \end{aligned}$$

and for *D+10*th day:

$$\begin{aligned} \begin{bmatrix} h^{13}_{01}&{}h^{13}_{12}&{}h^{13}_{23}\\ \end{bmatrix} \end{aligned}$$

Similarly, as described for Objective 1, the next iterations will take the same form. The only difference is the use of the vehicle count data as an input window sequence while for the output window sequence, the air pollutant data matrix is used.

**Figure 6:**
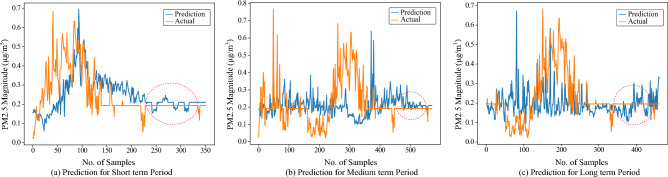
A comparison of prediction graphs (Blue) and actual graphs (Brown) for sensor 3 test data for *D*+1st (short term period), *D*+5th (medium term period) and *D*+10th (long term period) day. The prediction graph follows the trend of the actual values graph in all three cases. The straight line encircled by the red dotted circle shows the mean values replaced by the missing values.

## Results

In this section, we provide the results of all the experiments conducted in this work to achieve our objectives. Our proposed model comprises three interconnected layers of LSTM blocks, organized in a stacked LSTM configuration, employing the ’relu’ activation function. Specifically, the initial layer incorporated 100 LSTM blocks, while the subsequent two layers comprised 64 and 50 LSTM blocks, respectively. The run-time of our model for training 19 weeks (about 4 and a half months) of data over 100 epochs took 16.81 seconds, while, validation of the test data (03 weeks data) took only 0.21 seconds. All computations have been performed on a standard desktop system of model XPS 8950 with 32 GB of RAM and an Inter i9-12900K (24 CPUs) processor running at 3.2 GHz.

The proposed solutions are evaluated in terms of Root Mean Squared Error (RMSE), Mean Absolute Error (MAE), Mean Square Error (MSE), and Mean Relative Error (MRE) which are the most commonly used error metrics for evaluating the regression models. These metrics are defined in Eqs.  11, 12, 13, and 14, respectively. In the equations, $\hat{x}_{i}$ represents the predicted value, $x_{i}$ represents the actual value recorded by the sensors, and *n* is the number of samples in the dataset.

11 $$\begin{aligned}{} & {} \begin{aligned} RMSE = \sqrt{(1/n) \sum _{i = 1}^{n} (x_{i} - \hat{x}_{i})^{2}} \end{aligned} \end{aligned}$$

12 $$\begin{aligned}{} & {} \begin{aligned} MAE = (1/n) \sum _{i = 1}^{n} \left| x_{i} - \hat{x}_{i} \right| , \end{aligned} \end{aligned}$$

13 $$\begin{aligned}{} & {} \begin{aligned} \text {MSE} = \frac{1}{n} \sum _{i=1}^{n} (x_i - \hat{x}_i)^2 \end{aligned} \end{aligned}$$

14 $$\begin{aligned}{} & {} \begin{aligned} \text {MRE} = \frac{1}{n} \sum _{i=1}^{n} \left| \frac{x_i - \hat{x}_i}{x_i} \right| \times 100\% \end{aligned} \end{aligned}$$

### Objective 1 (AQI prediction)

#### AQI prediction using station data only

After the required pre-processing steps the sequenced input is fed into the model, which provides the required predictions. During the experiments, we observed that the predicted values are converging towards the pattern of the actual graph for all prediction periods (i.e., *D*+1st day, *D*+5th day, and *D*+10th) as shown in Fig. 6. Figure 6 provides the comparison of prediction graphs at station 3 for the next day (i.e., *D*+1st day), *D*+5th day, and *D*+10th day. However, as can be observed in the figure, the actual graph has a major portion as a straight line framed in red dotted lines, which is mainly due to the missing values replaced by the mean values.

Table 3 provides the results of the first objective showing the RMSE, MAE, MSE, and MRE values for *D*+1st, *D*+5th, and *D*+10th day at all stations. We note that each station is represented by the corresponding sensor. For example, sensor 1 represents station 1. Variations in the results of different sensors are due to different data records for every sensor, variations in data length, and different no. of missing values. As can be seen, overall better results are obtained in terms of RMSE, MAE, MSE, and MRE at all the stations. Overall, the lowest RMSE, MAE, and MSE values for *D*+1st, *D*+5th, and *D*+10th day are obtained at station/sensor 5.

**Table 3:** Error results for all the 10 sensors for Short, Medium, and Long Term Periods. Sensor 3 is the focus of the study and for comparative analysis for Objective 2 and other various models tested in this research work.

Stations	RMSE (PM2.5)			MAE (PM2.5)			MSE (PM2.5)			MRE (PM2.5)		
	D+1st	D+5th	D+10th	D+1st	D+5th	D+10th	D+1st	D+5th	D+10th	D+1st	D+5th	D+10th
Sensor 1	0.1253	0.1410	0.1467	0.093	0.121	0.127	0.0102	0.0204	0.0305	0.416	0.905	1.77
Sensor 2	0.1834	0.1610	0.1396	0.093	0.104	0.078	0.0196	0.0152	0.0246	0.380	0.208	0.338
Sensor 3	**0.1208**	**0.1097**	**0.069**	**0.082**	**0.083**	**0.045**	**0.0139**	**0.0184**	**0.0026**	**0.359**	**0.304**	**0.226**
Sensor 4	0.1312	0.1395	0.1623	0.077	0.066	0.116	0.0114	0.0174	0.0179	0.267	0.265	0.286
sensor 5	0.0192	0.0284	0.0314	0.018	0.019	0.029	0.0022	0.0007	0.0005	0.442	0.234	0.234
Sensor 6	0.0345	0.0406	0.0426	0.028	0.029	0.029	0.0011	0.0021	0.0006	0.906	1.22	1.30
Sensor 7	0.1713	0.1804	0.1905	0.094	0.072	0.101	0.0537	0.0389	0.0341	1.42	0.961	1.40
Sensor 8	0.033	0.0231	0.0373	0.015	0.012	0.015	0.0009	0.0009	0.001	0.204	0.210	0.244
Sensor 9	0.0733	0.0765	0.0759	0.053	0.060	0.056	0.0072	0.0096	0.0046	0.787	0.608	0.926
Sensor 10	0.069	0.0626	0.1099	0.050	0.052	0.065	0.0106	0.0081	0.0116	0.956	1.26	2.17

Significant values are in bold.

### Reflections on AQI prediction using station data only

In this study, we examined the forecasting of PM2.5 values for the short, medium, and long-term period, using a fixed *hour count* of 3 and *day count* of 4 while changing the *lookahead* variable from 1 to 5 and 10, respectively. Our results demonstrate the potential of the proposed model for predicting air pollutant levels and forecasting AQI.

#### AQI prediction using multiple stations data via a merit-based fusion scheme

In the third task, we conducted two different experiments to show the advantages of the fusion of data from multiple stations/sensors. In the first experiment, we trained individual models on data obtained from six different sensors/stations and predicted the air quality for the same stations (i.e., single input and single output). This way, we want to analyze the variations in the predictions of models trained on data from different stations. Here we take the leverage of the results obtained in Objective 1 in table 3. Significant variations can be observed in the results of these models for most of the target stations. These variations in the results provide the basis for our second experiment where we trained a single joint model on the data obtained from all the stations, which is then used to predict the air quality at each station.

**Table 4:** Experimental results of the fusion vs individual sensors as input: Results exhibit superior (highlighted) or comparable performance.

Inputs	Outputs	Forecast based on fusion input				Forecast based on individual sensors Input			
		RMSE	MAE	MSE	MRE	RMSE	MAE	MSE	MRE
Sensor 3, Sensor 7, Sensor 10, Sensor 1, Sensor 6, Sensor9	Sensor 3	0.1373	0.1028	0.021	0.7113	0.1208	0.082	0.0139	0.359
	**Sensor 7**	**0.1092**	**0.0853**	**0.0113**	2.0674	**0.1713**	**0.094**	**0.0537**	1.42
	Sensor 10	0.1109	0.0753	0.0134	2.054	0.069	0.050	0.0106	0.956
	**Sensor 1**	**0.0626**	**0.0453**	0.0221	**0.388**	**0.1253**	**0.093**	0.0102	**0.416**
	Sensor 6	0.0413	0.032	0.0015	1.1883	0.0345	0.028	0.0011	0.906
	**Sensor 9**	0.0891	0.0598	**0.0052**	0.9458	0.0733	0.053	**0.0072**	0.787

Significant values are in bold.

Table 4 shows the results of the second experiment where a single joint model has been trained on the data obtained from the six sensors/stations for the prediction of air quality at different regions. A comparison of the results, where the fusion of sensors is done and fed as an input to the model and the individual sensors as input to the model, shows that in most cases, the joint model has almost the same or better predictions compared to the individual models trained on the single station data.

### Reflections on AQI prediction using multiple stations data via a merit-based fusion scheme

In this investigation, we presented findings concerning the prediction of PM2.5 levels specifically for the subsequent day (referred to as D+1st day). The outcomes obtained provide support for the utilization of a single model trained on the complete array of city-installed sensors located across various points. This single generic model would facilitate predictions for distinct areas, thus superseding the need for separate models dedicated to individual sensors positioned at different locales.

### Objective 2 (periodic traffic-pollution patterns discovery)

**Figure 7:**
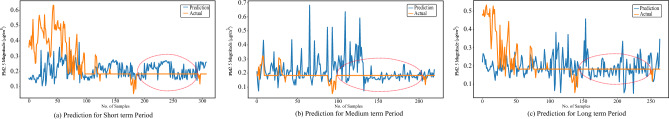
A comparison of prediction graphs (Blue) and actual graphs (Brown) for sensor 3 test data as an output and vehicle count from CCTV images as an input for *D*+1st (short term period), *D*+5th (medium term period) and *D*+10th (long term period) day. The straight line encircled by the red dotted circle shows the mean values replaced by the missing values.

Figure 7 shows the forecast results for the *D*+1st day (i.e. the next day), *D*+5th day, and *D*+10th day air pollutant values where the input of the model is the vehicle count from the images of the CCTV camera 9 and the forecast output is the PM2.5 values of station 3 (sensor 3). Some correlation can be observed in the graphs as the PM2.5 values increase with an increase in the number of vehicles.

The results of the experiment in terms of RMSE, MAE, MSE, and MRE for *D*+1st, *D*+5th and *D*+10th days are shown in Table 5. RMSE of 0.1267 for the next day, RMSE of 0.1236 for the 5th day, and RMSE of 0.0761 for the 10th day are observed as compared to the RMSE values of 0.1208 (D+1st), 0.1097 (D+5th) and 0.069 (D+10th) from Table 3 of sensor 3 which is the focus of the study. The results obtained are almost nearly equal in both cases. Results obtained through MAE, MSE, and MRE are also comparable.

**Table 5:** Error values for Objective 2. RMSE, MAE, MSE, and MRE error values show nearly same results for the training of the model on both sensor’s data (sensor 3) and vehicle count from CCTV images (camera 9) as compared to training on only the sensor’s data (sensor 3) in Table 3.

Objective 2 error Results	RMSE	MAE	MSE	MRE
Short term (D+1st day)	0.1267	0.0847	0.027	0.572
Medium-term (D+5th day)	0.1236	0.069	0.0167	0.3527
Long term (D+10th day)	0.0761	0.0563	0.0045	0.3066

**Figure 8:**
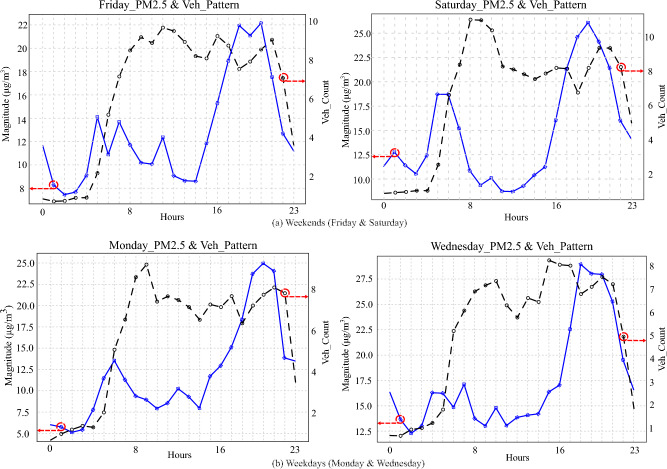
Daily pattern of PM2.5 concentration and public traffic, averaged over 05 months data: The pattern demonstrates the progressive accumulation of pollution from morning until evening, culminating in its peak during the evening hours, aligning with the heightened traffic levels observed throughout the daytime.

For our selected sensor 03 and camera 09, Fig. 8 shows patterns of PM2.5 concentration alongside traffic patterns for four days of the week averaged over 05 months of available data. Two days were taken on the weekends and two days taken as working days, i.e., Monday and Wednesday, in order to study the different patterns on weekends and normal weekdays. We have observed that traffic activity is highest during the daytime, whereas at midnight, very few vehicles are captured in CCTV images, mostly consisting of parked vehicles. Traffic activity gradually increases from 05:00 am, peaking at 09:00 am to 12:00 pm, and then stabilizes during the daytime with an abrupt decrease of transport from 09:00 pm to 11:00 pm. Analysis of image data reveals patterns of urban life activities of the public, with traffic activity being the lowest at midnight. The pattern of PM2.5 also shows consistency, whether it’s a weekend or a working day. PM2.5 concentration is always on the rise in the morning time and peaks in the evening time but drops back to lower values at midnight as PM2.5 particles accumulate with the increase in traffic vehicle activities throughout the day and drop to a minimum with the gradual decrease in traffic activities later in the day. Also, these patterns can also be taken as precautionary measures in favor of the public to educate them to avoid evening outdoor activities due to poor air quality.

### Reflections on periodic traffic-pollution patterns discovery

The outcomes derived from this study advocate for the incorporation of vehicle counts extracted from CCTV camera imagery as an input parameter for forecasting AQI across various temporal scenarios: short-term, medium-term, and long-term. This underscores the viability of utilizing CCTV traffic images as a viable alternative data source for AQI prediction purposes.

In the latest version of The Little Green Data Book^28^, 2017, two critical pollution indicators for PM2.5 exposure were mentioned. These are the Mean Annual exposure to PM2.5 pollution and the percentage of the total population exposed to PM2.5 pollution above WHO guideline values. In the case of Vietnam, both values have been found to be alarmingly high with the PM2.5 levels recorded to be 28 ($\mu$g/cu.m) and the percentage population exposed to the same is 100 percent. This indicator covers the entire country. In comparison, our model gives localized predictions of PM2.5 levels at different times of the day helping the authorities make timely decisions on providing public guidance on when to avoid outdoor activities, ensuring public health safety.

### AQI calculation

Once the air pollutant values are forecasted in Objective 1, the AQI is calculated using the procedures and index table provided by the US Environmental Protection Agency (EPA)^1^. The US EPA has provided a technical assistance document for the reporting of Daily Air Quality, which includes all the necessary details for calculating AQI. According to this document, the air quality is divided into 6 different categories according to the range of AQI based on pollutant values as shown in Table 6.

**Table 6:** AQI ranges and Air Quality Categories as per US Environmental Protection Agency (EPA)^1^.

Color	AQI range	Air quality description
Green	0 to 50	Good
Yellow	51 to 100	Moderate
Orange	101 to 150	Unhealthy for sensitive groups
Red	151 to 200	Unhealthy
Purple	201 to 300	Very unhealthy
Maroon	301 to 500	Hazardous

Table 7 provides the comparison of the predicted PM2.5 values and the corresponding AQI and air quality category against the actual PM2.5 and AQI values and the air quality category. As can be seen, in the majority of the cases our proposed solutions have accurately predicted the air quality category based on the predicted PM2.5 and AQI values, which shows the effectiveness of the proposed solution. There are also a few cases where the proposed solution made incorrect predictions as highlighted in yellow. In the incorrect cases, the difference in the forecasted values is small, however, when used for the calculation of AQI and categorized the air quality it changed the description of the air. Moreover, for the first two highlighted cases where the model is predicting Moderate air quality instead of Good, is a false negative and is acceptable in terms of public health. However, the third highlighted case where the model is predicting Moderate instead of Unhealthy for Sensitive Groups is a false positive which is not good for the public health experience.

**Table 7:** Comparison of Actual values of PM2.5 concentration, calculated AQI and Air categories alongside their corresponding predicted PM2.5 values, calculated AQI and air categories. Predicting Moderate level instead of Good is acceptable but predicting Moderate instead of Unhealthy for Sensitive Groups can be harmful to the public.

Task_1	Actual values			Predicted values		
	PM2.5	AQI	Category	PM2.5	AQI	Category
D+1	14.6	60.8	Moderate	12.9	53.7	Moderate
	14.8	61.6	Moderate	12.4	51.6	Moderate
	15.8	65.8	Moderate	15.2	63.3	Moderate
D+5th	14.4	60	Moderate	16.4	68.3	Moderate
	10.5	43.7	**Good**	13.3	55.4	***Moderate***
	7.9	32.9	**Good**	12.9	53.7	***Moderate***
D+10th	32.9	137	Unhealthy for sensitive groups	29	120	Unhealthy for sensitive groups
	24.7	102.9	*Unhealthy for sensitive groups*	17.7	73.75	***Moderate***
	14.4	60	Moderate	13.1	54.5	Moderate

Significance values are in bold, italic and bolditalic.

### Comparative analysis

For comparative analysis, we used the station/sensor’s data only tested it on different models, and recorded the results in order to show the effectiveness of the proposed LSTM-based solutions including all the variants over the conventional models namely Auto Regressive Moving Average (ARMA) and its updated version Auto-Regressive Integrated Moving Average (ARIMA). The contrast of outcomes is detailed in Table 8, revealing a notable enhancement in performance for the suggested approach across all iterations of the LSTM model, as compared to both ARIMA and ARMA methods. Notably, among the various LSTM iterations - namely Bi-directional LSTM, CNN LSTM, and ConvLSTM - the ConvLSTM variant stands out as the most proficient performer. Stacked LSTM exhibits comparatively inferior performance compared to other LSTM variants, while ARIMA outperforms ARMA. Through a comparison between ARIMA and Stacked LSTM outcomes, it becomes evident that Stacked LSTM demonstrates performance enhancements of 48%, 67%, and 173% for short, medium, and long-term periods, respectively. These findings collectively underscore the efficacy of the recommended LSTM framework.

**Table 8:** Results comparison between different variants of LSTM and conventional models i.e. ARMA and ARIMA. ConvLSTM produces the best results (highlighted in Green). Compared with Stacked LSTM, ARIMA results are lagging around 48%, 67% and 173% for the short, medium and long-term periods, respectively.

Stations	RMSE (PM2.5)			MAE (PM2.5)			MSE (PM2.5)			MRE (PM2.5)		
	D+1st	D+5th	D+10th	D+1st	D+5th	D+10th	D+1st	D+5th	D+10th	D+1st	D+5th	D+10th
ARMA	0.5243	0.5329	0.5378	0.5032	0.5162	0.5183	0.435	0.529	0.5132	2.723	1.651	1.589
ARIMA	0.1788	0.1833	0.1889	0.1598	0.1629	0.1643	0.1039	0.1135	0.1247	1.065	0.9531	0.6925
Stacked LSTM	0.1208	0.1097	0.069	0.032	0.083	0.045	0.0139	0.0184	0.0026	0.3592	0.3046	0.2262
Bi-directional LSTM	0.0294	0.0593	0.0454	0.0239	0.0377	0.0353	0.0122	0.0225	0.0038	0.355	0.3681	0.2946
CNN LSTM	0.0180	0.0527	0.0589	0.0086	0.0378	0.0442	0.0012	0.0187	0.0025	0.358	0.352	0.306
ConvLSTM	0.0135	0.0539	0.0518	0.0068	0.0389	0.0438	0.0118	0.0192	0.0035	0.351	0.363	0.258

### Summary and lessons learned

The proposed solution for forecasting in Objective 1 i.e. AQI Prediction, performed well as compared to the conventional time series analysis models. For object detection in images in Objective 2 i.e. Periodic traffic-pollution patterns discovery, initially, we used YOLOv5 but the results were not good. The model missed a few vehicles in most of the images, which resulted in a lower count of the vehicles to be fed into the model. To overcome this issue, we employed YOLOv8x which significantly improved the vehicle detection capabilities of the framework.

During the analysis, we observed that data must exhibit seasonality and consistency for better forecasting of AQI. In order to enhance prediction accuracy, it is essential to have access to an extended historical dataset that encompasses patterns occurring throughout the entire year. However, it is crucial to remain cognizant of the insights presented by^29^, which underscore the limitations and potential drawbacks associated with the adoption of Big Data. Their research cautions against the presumption that Big Data can entirely supplant traditional data sources, as this does not inherently assure greater accuracy or reliability. Additionally, the text emphasizes the valuable role of traditional “small data,” noting that it can provide distinctive insights that may remain elusive when exclusively relying on big data. The narrative advocates for the integration of both conventional and novel data reservoirs. These two points of having small data and integration of new sources with conventional resources are the key strengths of our work. We also found dealing with missing logs very challenging because of faulty sensors and CCTV cameras. This offline time of sensors and CCTV cameras has an impact on the prediction results and disables the model to completely learn the urban activities. Upon analyzing the actual data against the predicted data, we noticed that the error tends to rise for larger values. Nevertheless, the occurrence of these higher values is so infrequent that it does not significantly influence the predictive outcomes of our model.

Moreover, in the task of AQI prediction using Multiple sensor data via a merit-based fusion scheme, a joint model by combining data from multiple stations/sensors for training is more effective compared to the individual models trained on single station/sensor data as these sensors may compensate for the missing values from some of the other sensors. Additionally, adopting this approach would enable the utilization of a unified model, eliminating the necessity for employing distinct models corresponding to different geographical regions.

## Conclusion and future work

This work is focused on developing a comprehensive forecasting framework for air quality by utilizing real-time data with real-time issues from multiple sources, including air pollutant sensors and CCTV cameras. The proposed framework forecasts air quality over short, medium, and long-term periods. our investigation delved into the effectiveness of a consolidated model trained on sensors deployed across diverse locations. This analysis highlights the feasibility of employing a singular generic model, eliminating the need for individual models corresponding to each sensor installation. Furthermore, We also explored the potential of CCTV cameras as an alternative input for predicting air quality. By detecting and counting vehicles in the CCTV images, we aimed to establish a correlation between vehicle count and air pollutant values. Our analysis led us to the conclusion that the vehicle count attribute derived from CCTV images holds significant importance in contrast to data from air pollutant sensors, particularly in the context of air pollutant (PM2.5) prediction. With this in mind, the integration of vehicle count data from CCTV images presents a promising alternative for predicting air quality. During the experiments and analysis, we evaluated LSTM and its variants as compared to the ML algorithms namely ARMA and ARIMA, and demonstrated their predictive capabilities by obtaining an improvement of 48%, 67%, and 173% for short-term, medium-term, and long-term periods, respectively, of Stacked LSTM over ARIMA.

In the future, we aim to assess the potential enhancements that transformers, when employed as a predictive model, could bring to existing methodology. We also aim to investigate the extent of performance improvement achievable through the involvement of various features from weather stations data like humidity, temperature, wind speed, pressure, rainfall, and dew point, etc. and from CCTV traffic images like a rainy day, sunny day, model and make a year of the vehicle and its emission rates, etc.

## Data Availability

The datasets used in this work could be downloaded from the source cited in the paper while the additional information is available from the corresponding author on reasonable request.
